# Randomized Phase II Trial of Triapine-Cisplatin-Radiotherapy for Locally Advanced Stage Uterine Cervix or Vaginal Cancers

**DOI:** 10.3389/fonc.2019.01067

**Published:** 2019-10-15

**Authors:** Charles A. Kunos, Stephen J. Andrews, Kathleen N. Moore, Hye Sook Chon, S. Percy Ivy

**Affiliations:** ^1^Cancer Therapy Evaluation Program, National Cancer Institute, Bethesda, MD, United States; ^2^SummaHealth Cooper Cancer Center, Akron, OH, United States; ^3^University of Oklahoma Stephenson Cancer Center, Oklahoma City, OK, United States; ^4^H. Lee Moffitt Cancer & Research Institute, Tampa, FL, United States

**Keywords:** triapine, cisplatin, radiotherapy, cervical cancer, uterine cervix cancer, randomized phase II

## Abstract

Uterine cervix or vaginal cancers have inherent overactivity of ribonucleotide reductase (RNR), making these cancers rational targets for therapy based on interruption of cisplatin-radiotherapy-induced DNA damage repair. We conducted a pilot, open-label randomized phase II trial to evaluate the efficacy and safety of cisplatin-radiotherapy with or without triapine, a small molecule with RNR-inhibitory activity, in patients with advanced-stage uterine cervix or vaginal cancers (NCT01835171), as a lead in to a randomized phase III study (NCT02466971). A total of 26 women were randomly assigned to receive 6 weeks of daily radiotherapy followed by brachytherapy (80 Gy) and once-weekly cisplatin (40 mg m^−2^)—with or without three-times weekly intravenous triapine (25 mg m^−2^)—in one 56-days cycle. Primary end points were metabolic complete response by positron emission tomography and safety. Additional end points included the rate of clinical response, rate of methemoglobinemia, and progression-free survival. The addition of triapine to cisplatin-radiotherapy improved the rate of metabolic complete response from 69 to 92% (*P* = 0.32) and raised the 3-year progression-free survival estimate from 77 to 92% (hazard ratio for progression, 0.30; *P* = 0.27). The most frequent grade 3 or 4 adverse events in either treatment group included reversible leukopenia, neutropenia, fatigue, or electrolyte abnormalities. No significant differences were seen between the two groups in the rate of adverse events. Symptomatic methemoglobinemia was not encountered after triapine infusion. In conclusion, the addition of triapine to cisplatin-radiotherapy improved the rate of metabolic complete response in patients with advanced-stage uterine cervix or vaginal cancers without significant toxicity. A phase III trial adequately powered to evaluate progression-free and overall survival is underway (NCT02466971).

## Introduction

Uterine cervix and vaginal cancers forecast as the fourth most common any-type cancers and will likely be the fourth leading cause of cancer-related death in women worldwide in 2020 (1). Nearly 36% of new uterine cervix cancer cases in American women are initially disease staged as regionally advanced, meaning the disease has spread from the uterine cervix to abdominopelvic lymph nodes or nearby visceral organs (2). An overactive ribonucleotide reductase (RNR) is the hallmark molecular driver in more than 80 percent of uterine cervix cancers (3–7). Women with regionally advanced-stage disease undergo 5-days radiation (180 cGy per daily fraction) repeated over 5½ weeks along with once weekly cisplatin chemotherapy infusions (40 mg m^−2^) followed by intracavitary brachytherapy (8–10). A disease response rate of 60 percent is expected (11, 12). A 36-month (3-year) survival rate for such treated patients is 69 percent (8–10).

One unifying approach to radiochemotherapy for uterine cervix cancer has been the pharmacologic inhibition of RNR (13, 14). In dormant (G_0_-phase) or resting (G_1_-phase) cells, when levels of RNR subunit proteins are low, enzyme activity, and therefore nucleotide output, is minimal (15). In cancer cells driven to replicate (high S-G_2_-phase), RNR subunit proteins are (over)expressed so that *de novo* nucleotide output is greatest—an activity mitigated by intrinsic feedback allosteric sites in RNR (16). Allosteric sites detect overall nucleotide concentration in cells and balance *de novo* nucleotide production in a way that drugs like 5-fluorouracil and gemcitabine can interfere (13, 14). When RNR is overactive, DNA damage repair is efficient and impacts downstream prosurvival cell fate decisions (17–20). Nucleotide pool expansion by RNR after DNA damage help cells survive. Such observations provide an attractive rationale for the clinical development of RNR inhibitors in uterine cervix cancers with unchecked RNR. Triapine (3-aminopyridine-2-carboxaldehyde thiosemicarbazone) is one such RNR inhibitor with well-characterized antiproliferative effects. In preclinical studies, triapine blocked deoxynucleotide output by RNR after DNA damage, protracted cell cycle arrest at the G_1_-S-phase restriction checkpoint, and led to unreconciled γH2AX foci (i.e., phosphorylated histones flanking DNA double-strand breaks) labeling unfixed DNA damage—all disruptive to normal RNR prosurvival functions (17–20). However, triapine monotherapy and triapine-cisplatin combination studies showed no substantial clinical activity (21–23).

Pharmacologic inhibition of RNR by hydroxyurea (20, 24) or by triapine (6, 7) during radiotherapy in women with regionally advanced-stage uterine cervix cancers has been studied with clinical benefit. An RNR inhibitor-cisplatin-radiotherapy combination resulted in an 87 percent rate of response in the phase II setting (25) and 3-years survival estimate of 68 percent in a phase III clinical trial (10). A triapine-cisplatin-radiotherapy combination resulted in a 96 percent rate of response in a single-arm phase II trial (7). Since there remains significant unmet therapeutic needs in women with regionally advanced-stage uterine cervix or vaginal cancers, we designed a randomized phase II trial to evaluate whether intravenous triapine could inhibit RNR and potentiate the antitumor effects of cisplatin-radiotherapy for improved survival outcomes within acceptable levels of toxicity.

## Materials and Methods

### Patients

Inclusion criteria for the study were female sex, an age of 18 years or older, and a histologically-documented diagnosis of stage IB2-IVA uterine cervix cancer, or stage II-IVA vaginal cancer, with measurable disease. Other inclusion criteria were Gynecologic Oncology Group performance status of 0, 1, or 2; no uncontrolled intercurrent illness; no other active invasive malignancies; no prior treatment; and adequate bone marrow, hepatic, and renal function. Central nervous system metastases were not permitted. A patient diagnosed with uncontrolled diabetes mellitus (fasting blood glucose > 200 mg/dL) was not permitted due to anticipated high glucose level interference in the conduct of ^18^F-FDG-positron emission tomography assessments. A patient with known glucose-6-phosphate dehydrogenase deficiency (G6PD) was not permitted due to an inability to administer the antidote methylene blue for triapine-related methemoglobinemia (26). At the time of study design, it was unknown whether triapine would interfere in a drug-drug interaction with combination antiretroviral therapy, and so, a patient with known human immunodeficiency virus infection was not permitted.

All patients provided written informed consent according to the Declaration of Helsinki before enrollment. Histopathology and all clinical laboratory tests were done according to each institution's standards.

### Study Design

The study was approved by the institutional review boards of all participating institutions. The study was sponsored by the National Cancer Institute (NCI) Cancer Therapy Evaluation Program (CTEP). The study was designed by the principal academic investigator in collaboration with NCI CTEP. Data collection and analysis were performed quarterly by Clinical Data Update System (CDUS) in collaboration with the sponsor. The principal academic investigator and accrual site investigators in collaboration with NCI CTEP vouch for the completeness and accuracy of the data, the data analyses, and the fidelity of this report for the NCI #9434 study protocol. The article was written by the principal academic investigator with editorial assistance provided by the sponsor. The article was reviewed by all coauthors and the sponsor.

This multicenter, open-label, randomized, phase II study was conducted at four sites. Patients were recruited from October 2013 through November 2015. Each center must have had a qualified and certified positron emission tomography scanner by the American College of Radiology Imaging Network (ACRIN) prior to enrollment of any patient onto protocol treatment. All eligible patients were randomly assigned, in a 1:1 ratio, to receive cisplatin plus radiotherapy and brachytherapy, either alone (the cisplatin-radiotherapy-alone group) or in combination with triapine (the triapine group). Assignment to treatment groups was conducted by means of a random allocation system at the coordinating cancer center. Randomization was not stratified according to study center but was stratified by anticipated brachytherapy technique (low-dose-rate vs. high-dose-rate). Treatment was to begin within 5 days of randomization.

The primary end point was post-therapy 3-month ^18^F-FDG-positron emission tomography and computed tomography response by European Organization for Research and Treatment of Cancer (EORTC) and NCI guidelines (27, 28). Secondary end points included post-therapy clinical response assessment by Response Evaluation Criteria in Solid Tumors (RECIST) version 1.1, progression-free and overall survival, peripheral venous blood methemoglobin proportion, as well as safety and tolerability of treatment by Common Toxicity Criteria for Adverse Events (CTCAE) version 4.0. Progression-free survival was defined as the time from randomization to confirmation of disease progression or death. Overall survival was defined as the time from randomization until the date of death. A cost analysis for a single triapine infusion was not a prespecified endpoint but was computed to investigate the potential impact of triapine cost on treatment compliance (as an oral triapine formulation is being studied in this same patient population, clinicaltrials.gov number NCT02595879).

The NCI #9434 protocol was amended in June 2015 to transition the trial to the NCI National Clinical Trials Network (NCTN) for accelerated patient accrual (clinicaltrials.gov number, NCT02466971) when other trial data showed substantial uterine cervix cancer response to triapine-cisplatin-radiotherapy (7) and for a change in primary end point of progression-free survival. We write this article as the final report of clinical outcomes and safety for 26 patients recruited to NCI protocol #9434 (obsolete clinicaltrials.gov number, NCT01835171).

### Treatment

Patients received radiotherapy as follows: within one 56 ± 3-days period, external beam 4-field pelvic radiotherapy (180 cGy/day) was given over 5 consecutive days (Monday through Friday) repeated for 5 consecutive weeks (25 treatments); followed by external beam anteroposterior-posteroanterior opposed parametrial boost (180 cGy/day) over 3 consecutive days (3 treatments) in a sixth week; followed by one or two low-dose-rate (total cumulative dose > 8,000 cGy) or five high-dose-rate (total cumulative dose > 7,500 cGy) intracavitary brachytherapy implants. Interstitial brachytherapy was permitted if intracavitary brachytherapy was not suitable as determine by the treating radiation oncologist and after an approved pretreatment brachytherapy plan review by the principal academic investigator.

Patients received chemotherapy as follows: within one 42-days period, on day 2 (Tuesday) of each of 6 weeks, intravenous cisplatin (40 mg m^−2^, 70 mg maximum) over a 90-min period after radiotherapy using non-aluminum administration sets accompanied by pre-therapy and post-therapy normal saline and antiemetic prophylaxis. This regimen was administered either alone or together with intravenous triapine (25 mg m^−2^) over a 90-min period after radiotherapy on days 1, 3, and 5 (Monday, Wednesday, Friday) of each of the first 5 weeks, or with make-up infusion(s) in the sixth week (meaning, 15 total infusions for the one cycle of treatment). Chemotherapy was not given during brachytherapy.

### Assessment

Tumor response was based on investigator assessment of target and non-target lesions by clinical examination at 1-month post-therapy and every 3-month post-therapy or by means of ^18^F-FDG-positron emission tomography and computed tomography at 3- and 6-month post-therapy, in the absence of clinically evident disease progression. Tumor measurements according to RECIST version 1.1, were used to evaluate clinical tumor response and to establish disease progression (29).

Safety was assessed with the use of standard clinical and laboratory tests (hematologic tests, blood chemical tests, or methemoglobin venous blood gas throughout the study period until 30 days after the last dose of a study drug was administered. Optional methemoglobin proportions (or the ratio of methemoglobin to normal hemoglobin) were acquired pre-therapy, 1, 3, 5, 6, 7, and 24 h after triapine infusion. On trial, the triapine antidote methylene blue was to be available at treating sites during triapine infusion (26). Adverse event grades were defined by CTCAE version 4.0. Serious adverse events were monitored and reported to NCI CTEP by the primary investigator at each cancer center. A Data and Safety Monitoring Committee (DSMC) at the coordinating center monitored this study following NCI CTEP guidelines.

### Evaluation of Clinical Activity and Statistical Analysis

**Figure 1:**
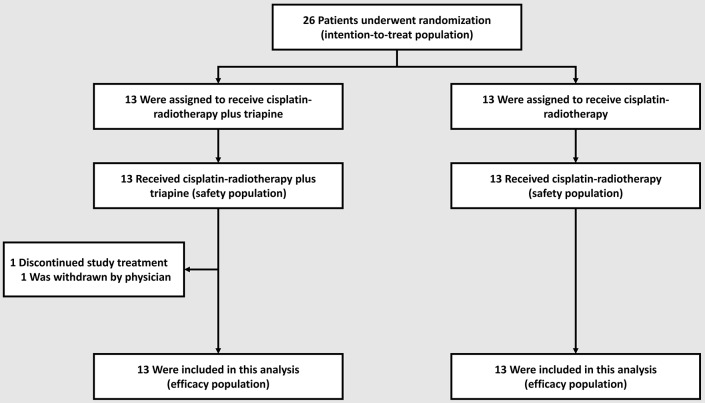
Enrollment, randomization, and follow-up of the study patients.

The primary objective of the trial was to estimate the metabolic complete response rate in the triapine group. The study team calculated that with a sample size of 33 patients per group, assuming that the observed rate of metabolic complete response in the triapine group was about 0.90 [or 90% (7)], the half-width of the exact 90% binomial confidence interval would be approximately equal to 0.09. In particular, for an observed rate of metabolic complete response of 0.90, the exact 90% binomial confidence interval was 0.79–0.97. The anticipated rate of metabolic complete response in the cisplatin-radiotherapy-alone group was about 0.60 [or 60% (3)]. If the rate of metabolic complete response in the triapine group was 0.886 or greater, then on the basis of a one-sided test of equality of proportions at the 10% level of significance, the trial would have a power of at least 80% to detect an increase from the rate of metabolic complete response of 0.60 in the cisplatin-radiotherapy alone group. The flow of patients through enrollment, randomization, and follow-up is depicted in Figure 1. Given the June 2015 amendment to transition this study to the NCI NCTN, a full complement of patient accrual was not achieved.

NCI has set forth guidelines for PET/CT response assessment (28)—a metabolic complete response was absence of abnormal FDG uptake at sites of abnormal FDG uptake noted on the baseline scan; partial metabolic response was 15–25% reduction in tumor FDG uptake; stable metabolic response ranged between <15% reduction or ≤ 25% gain in tumor FDG uptake; and progressive metabolic disease was labeled as >25% gain in tumor FDG uptake or appearance of new FDG uptake in metastatic lesions. A computed tomography scan was co-acquired for anatomic detail. To overcome interpretive challenges of physiologic FDG uptake in irradiated central pelvic tumor, a ratio of 3-month post-therapy to baseline pre-therapy maximum FDG standard uptake value (SUV) in tumor was computed, as done elsewhere (3, 7). A benchmark threshold ratio of 0.33 was applied for metabolic complete response (3). Corresponding clinical tumor response by RECIST 1.1 was recorded.

Descriptive statistics are provided for each trial group (Table 1). For this article, only metabolic or clinical complete responses were considered as the useful efficacy measure of clinical activity (Table 2), and thus, two-way contingency table statistics are provided (α = 0.05). Efficacy end points of progression-free and overall survival were estimated, and 95% confidence intervals were calculated, by means of the Cox proportional hazards method (Table 2). The distributions of overall survival in the two groups were not compared due to too few events in each group. Adverse events and serious adverse events were tabulated according to trial group and the CTCAE version 4.0 categorization and preferred terms (Table 3).

**Table 1:** Baseline characteristics of the study patients, according to treatment group.

**Characteristics**	**Cisplatin-radiotherapy and triapine (*N* = 13)**	**Cisplatin-radiotherapy (*N* = 13)**
Female sex—no. (%)	13 (100)	13 (100)
**Age—year**		
Median	50	55
Range	29–70	29–64
Race—no. (%)^*^		
White	8 (62)	11 (85)
Black or African ancestry	1 (8)	1 (8)
Asian	2 (16)	0
American Indian or Alaska Native	1 (8)	1 (8)
Native Hawaiian or Other Pacific Islander	1 (8)	0
Ethnicity—no. (%)^*^		
Not Hispanic or Latina	11 (85)	13 (100)
Hispanic or Latina	2 (16)	0
**GOG performance status—no. (%)**^†^		
0	13 (100)	12 (92)
1	0	1 (8)
2	0	0
**Disease stage**		
IB2	2 (16)	4 (31)
IIA	3 (23)	1 (8)
IIB	6 (46)	6 (46)
IIIA	0	0
IIIB	2 (16)	1 (8)
IVA	0	1 (8)
**Disease histopathology**		
Squamous cell carcinoma	8 (62)	11 (85)
Adenocarcinoma	3 (23)	0
Adenosqamous cell carcinoma	0	0
Vaginal squamous cell carcinoma	2 (16)	2 (16)

* *Race and Ethnicity were self-reported*.

† *The Gynecologic Oncology Group (GOG) performance status reflects the daily-living abilities of the patient, on a scale of 0 (fully active without symptoms) to 5 (dead)*.

**Table 2:** Summary of efficacy measures in the intention-to-treat population^*^.

**Outcome**	**Cisplatin-radiotherapy and triapine (*N* = 13)**	**Cisplatin-radiotherapy (*N* = 13)**	***P*-value**
Progression-free survival			
3-years estimate (95% CI)	92% (54–99%)	77% (44–92%)	0.27
Hazard ratio (95% CI)	0.30 (0.03–2.94)		
Overall rate of response—no. (%)	13 (100)	10 (77)	0.22
Best overall clinical response—no. (%)^†^			0.16
Complete response	12 (92)	8 (62)	
Partial response	1 (8)	2 (16)	
Progressive disease	0	3 (23)	
Best overall PET/CT response—no. (%)			0.58
Complete response	12 (92)	9 (69)	
Partial response	1 (8)	1 (8)	
Progressive disease	0	1 (8)	
Not able to be evaluated	0	2 (16)	

* *CI denotes confidence interval*.

† *Patients for whom best overall response could be evaluated had completed at least 1 day of treatment and had undergone both baseline and post-treatment assessment of physical tumor size*.

‡ *Patients for whom best overall positron emission tomography/computed tomography (PET/CT) response could be evaluated had completed at least 1 day of treatment and had undergone both baseline and post-treatment assessment of metabolic response*.

**Table 3:** Adverse events in the safety population^*^.

**Event**	**Cisplatin-radiotherapy and triapine (*****N*** **=** **13)**					**Cisplatin-radiotherapy (*****N*** **=** **13)**				
	**Grade**					**Grade**				
	**1**	**2**	**3**	**4**	**5**	**1**	**2**	**3**	**4**	**5**
	***Number of patients (percent)***									
Allergy/Immunology	0	0	0	0	0	0	0	0	0	0
Blood/Bone marrow (other)	0	0	0	0	0	0	0	0	0	0
Anemia	1 (8)	7 (54)	1 (8)	0	0	3 (23)	4 (31)	1 (8)	0	0
Neutropenia	3 (23)	2 (15)	2 (15)	0	0	0	4 (31)	1 (8)	0	0
Leukopenia	3 (23)	1 (8)	2 (15)	3 (23)	0	1 (8)	0	4 (31)	0	0
Thrombocytopenia	2 (15)	0	0	0	0	5 (38)	2 (15)	0	0	0
Cardiovascular (other)	0	0	0	0	0	0	0	0	0	0
Hypertension	0	0	1 (8)	0	0	0	1 (8)	1 (8)	0	0
Tachycardia	0	0	0	0	0	0	1 (8)	0	0	0
Constitutional (other)	0	0	0	0	0	2 (15)	0	0	0	0
Anxiety	1 (8)	0	1 (8)	0	0	0	0	0	0	0
Fatigue	7 (54)	2 (15)	2 (15)	0	0	4 (31)	6 (46)	0	0	0
Headache	0	1 (8)	0	0	0	0	0	0	0	0
Insomnia	0	1 (8)	0	0	0	1 (8)	0	0	0	0
Dermatology/Skin (dermatitis)	3 (23)	1 (8)	0	0	0	5 (38)	0	1 (8)	0	0
Endocrine/Special Senses (other)	0	0	0	0	0	0	0	0	0	0
Tinnitus	0	0	0	0	0	0	0	0	0	0
Hearing Loss	0	0	0	0	0	0	0	0	0	0
Blurred Vision	1 (8)	0	0	0	0	1 (8)	0	0	0	0
Gastrointestinal (other)	0	0	0	0	0	1 (8)	0	0	0	0
Anorexia	4 (31)	1 (8)	0	0	0	3 (23)	0	0	0	0
Diarrhea	8 (62)	4 (31)	1 (8)	0	0	9 (69)	2 (15)	0	0	0
Emesis	0	0	1 (8)	0	0	5 (38)	1 (8)	0	0	0
Nausea	6 (46)	2 (15)	1 (8)	0	0	10 (77)	2 (15)	0	0	0
Infection (urinary tract)	2 (15)	2 (15)	0	0	0	2 (15)	3 (23)	0	0	0
Metabolic/Nutritional (other)	6 (46)	0	0	0	0	4 (31)	2 (15)	1 (8)	0	0
Creatinine increased	1 (8)	0	0	0	0	1 (8)	0	0	0	0
Hypoalbuminemia	5 (38)	1 (8)	0	0	0	2 (15)	1 (8)	0	0	0
Hypokalemia	2 (15)	0	1 (8)	0	0	4 (31)	0	2 (15)	0	0
Hypomagnesemia	3 (23)	0	0	0	0	4 (31)	0	2 (15)	0	0
Hyponatremia	2 (15)	0	0	0	0	3 (23)	0	0	0	0
Musculoskeletal	0	0	0	0	0	0	0	0	0	0
Neuropathy (peripheral)	5 (38)	0	0	0	0	2 (15)	0	0	0	0
Pain (any)	7 (54)	4 (31)	0	0	0	6 (46)	3 (23)	1 (8)	0	0
Respiratory System (other)	0	0	0	0	0	0	0	0	0	0
Dyspnea	3 (23)	2 (15)	0	0	0	2 (15)	1 (8)	0	0	0
Hypoxia	0	0	0	0	0	0	0	0	0	0
Thromboembolic event	0	0	0	0	0	0	1 (8)	0	0	0
Renal/genitourinary (cystitis)	2 (15)	0	0	0	0	1 (8)	0	0	0	0
Sexual/reproductive function	4 (31)	1 (8)	0	0	0	6 (46)	0	0	0	0
Any event	13 (100)	13 (100)	9 (69)	3 (23)	0	13 (100)	13 (100)	11 (85)	0	0

* *Patients may have had more than one adverse event. The safety population included all patients who received at least on dose of a study drug*.

## Results

### Patients

Between October 2013 and November 2015, 26 patients were randomly allocated to a treatment group−13 to the cisplatin-radiotherapy-alone group and 13 to the triapine group. All 26 (100%) patient received at least one assigned treatment and were included in the safety analysis: one patient in the triapine group had an adverse event after the second triapine infusion, was withdrawn from protocol treatment by the treating physician, and completed cisplatin-radiotherapy alone (Figure 1). As of the date of data cutoff, May 5, 2019, all patients have completed treatment; no patient analyzed here contributes data to the amended NCTN trial.

Overall, the two treatment groups were well-balanced for baseline patient characteristics (Table 1). All 26 patients (100%) received their randomly allocated treatment as their first line of anticancer therapy. Radiotherapy was completed in a median 51 days (25–75% quartile: 48–53 days). Radiotherapy quality assurance review found that one patient in the triapine group underwent external beam boost radiotherapy without brachytherapy, two patients in the triapine group and one patient in the cisplatin-radiotherapy alone group underwent radiotherapy and brachytherapy without mandatory parametrial boost. Twelve (92%) of 13 patients in the triapine group received the intended 15 triapine infusions. Twenty-five (96%) of 26 patients received at least four cisplatin infusions, consistent with prior experience (9).

### Efficacy

For the intention-to-treat groups, the rate of clinical complete response was 92% (12 of 13 patients) in the triapine group and 62% (8 of 13 patients) in the cisplatin-radiotherapy-alone group (*P* = 0.18). The overall rate of response was 100% (13 of 13 patients) in the triapine group and 77% (10 of 13 patients) in the cisplatin-radiotherapy-alone group (*P* = 0.22) (Table 2).

For patients who received at least one infusion of a study agent and underwent both baseline and post-therapy ^18^F-FDG-PET/CT assessments, the rate of metabolic complete response was 92% (12 of 13 patients) in the triapine group and 82% (9 of 11 patients) in the cisplatin-radiotherapy-alone group (*P* = 0.58). One patient in the triapine group achieved a metabolic partial response after receiving only two infusions of the study agent, and then, completing cisplatin-radiotherapy alone. In the cisplatin-radiotherapy-alone group, one patient achieved a metabolic partial response, one had disease progression by the 3-month assessment, and two had disease progression precluding post-therapy ^18^F-FDG-PET/CT assessment.

Median pretherapy ^18^F-FDG-PET standard uptake value in a uterine cervix or vaginal tumor was 13.7 (25–75% quartile: 8.7–16.7); the corresponding median 3-month post-therapy ^18^F-FDG-PET standard uptake value was 3.0 (25–75% quartile: 0.0–4.0). Applying a more stringent criteria of a post-therapy-to-pre-therapy ^18^F-FDG-PET standard uptake value ratio <0.33, the complete metabolic response rate of the primary tumor was 88% (21 of 24). The median SUV ratio was 0.14 (25–75% quartile: 0.00–0.25). The median SUV ratio was 0.09 in the triapine group and 0.14 in the cisplatin-radiotherapy-alone group (*P* = 0.64).

A 3-year estimate for progression-free survival was 92% in the triapine group and 77% in the cisplatin-radiotherapy-alone group (hazard ratio for disease progression with triapine, 0.30; 95% confidence interval [CI]: 0.03–2.94, *P* = 0.27) (Table 2). Median progression-free survival was not reached in either group.

Median follow-up was 40 months (25–75% quartile: 22–53 months). A single confirmed non-cancer cause of death in the triapine group occurred during the study period for a 3-year estimate for overall survival of 92% (95% CI: 54–99%). In the cisplatin-radiotherapy-alone group, one patient one had disease progression by the 3-month assessment and is alive with metastatic disease after second-line therapy; two had disease progression precluding post-therapy ^18^F-FDG-PET/CT assessment and vital status remained unconfirmed due to patient relocation and loss to follow-up.

### Safety

Table 3 lists the most common adverse events in the safety population. The most frequent adverse events included grade 1 or 2 fatigue, nausea, diarrhea, and electrolyte abnormalities; grade 2 or 3 anemia and neutropenia; and grade 3 or 4 leukopenia. The incidence of grade 3 or 4 adverse events was 92% (12 of 13) in the triapine group and 85% (11 of 13) in the cisplatin-radiotherapy-alone group (*P* = 1.0); these events mostly involved neutropenia or leukopenia. The rate of grade 4 leukopenia was more than five percent higher in the triapine group than in the cisplatin-radiotherapy-alone group, but no significant differences were observed in the frequency of any adverse event between the two treatment groups. A single patient in the triapine group discontinued treatment because of adverse events. The dose of triapine was never reduced in the triapine group. The median number of triapine infusions administered was 15 in the triapine group and the median number of cisplatin infusions was five in the cisplatin-radiotherapy-alone group. No fatal adverse events occurred during study treatment.

Normal methemoglobin proportion is one to three percent (26). Methemoglobinemia is a known triapine-related adverse event (26). For patients who received at least one infusion of triapine and underwent methemoglobin assessment, the peak methemoglobin proportion encountered on trial was 1.3% 2 h after the start of triapine in one patient (a rise from a pretherapy methemoglobin proportion of 0.1%). The average (standard deviation) methemoglobin proportion before triapine infusion was 0.23% (0.12%) and after triapine infusion was 0.48% (0.41%), a 2-fold increase. No patient on trial had the methylene blue antidote administered after any triapine infusion.

### Cost Deterministic Results

Total drug costs for triapine were not tabulated as the agent was provided free-of-charge by NCI CTEP as an investigational new drug (IND 68338) in a uterine cervix cancer trial with therapeutic intent as a primary objective. Costs for venipuncture agent administration, physical examination, complete blood count with differential, blood electrolyte assessment, infusion chair time, adverse event monitoring, and nursing or regulatory procedure expenses were $734.18 on average per patient per week on treatment. A total 6-weeks cost for triapine administration was $4,405.09 on average per patient. Costs for post-therapy physician visits, monitoring for clinical disease progression, and nursing or regulatory procedure or time expenses were $3,948.80 on average per enrolled patient. The total drug administration cost was $8,353.89 on average. For patients who received at least one infusion of a study agent and the payment method was known, the payer mix on behalf of the patients in the triapine group was private insurance (*n* = 7), federal insurance (*n* = 1), or Medicaid (*n* = 3).

## Discussion

This open-label randomized phase II trial showed that the addition of triapine to cisplatin-radiotherapy improved measures of efficacy, including the rate of metabolic complete response, clinical complete response, and progression-free survival in women with advanced-stage uterine cervix or vaginal cancers.

The rate of metabolic complete response was selected as the primary end point for this study, rather than the more commonly used progression-free survival. The rate of metabolic complete response was selected on the basis of the hypothesis that triapine may exert more profound cytotoxic effects in a cancer harboring overactive ribonucleotide reductase, resulting in more substantial tumor regression earlier (3-month post-therapy) than could be detected by standard clinical assessment (3-year post-therapy). For this reason, metabolic complete response was regarded by NCI and by ACRIN as clinically meaningful in assessing the antitumor activity of triapine. The metabolic complete response rate by SUV uptake ratio in the triapine group of 92% is consistent with a long-term 95% metabolic complete response rate seen in previous triapine-cisplatin-radiotherapy clinical trials (30). The generalizability and transferability of the metabolic complete response findings in this study are limited; the on-going phase III trial of triapine-cisplatin-radiotherapy is adequately powered to evaluate better the metabolic complete response in uterine cervix or vaginal cancers (NCT02466971). So far, the clinical activity of triapine-cisplatin-radiotherapy has translated to a non-significant 15 percent improvement in 3-year progression-free survival.

A triapine-cisplatin-radiotherapy combination has been evaluated in several studies of advanced-stage uterine cervix and vaginal cancer clinical trials (6, 7). In this randomized trial, as before, triapine was given on three-times weekly schedule, in close proximity to external beam radiotherapy, to take advantage of possible synergy among the DNA damage induced by radiation and protracted repair of DNA damage by an inhibited ribonucleotide reductase. The overall rate of response in the cisplatin-radiotherapy-alone group (77%) was similar to the rate described in prior studies (range, 52–88%) (11, 12). The addition of triapine to cisplatin-radiotherapy increased the overall rate of response to 100% (*P* = 0.22), suggesting that triapine may overcome intrinsic resistance of an overactive ribonucleotide reductase that facilitates DNA damage repair. Most patients eventually did not have disease progression while receiving triapine-cisplatin-radiotherapy suggesting limited acquired resistance to triapine.

The triapine group had no significant increase in toxicity as compared to cisplatin-radiotherapy-alone group. The similar safety profiles in the two groups may be attributable to specificity in the targeting of uterine cervix or vaginal cancer cells with overactive ribonucleotide reductase, which spares normal, ribonucleotide reductase–proficient cells. Differences in the risk of adverse events between the two groups were minimal. Methemoglobin proportion did not rise to a previously observed symptomatic toxicity (which is a 20% methemoglobin proportion) and no methylene blue antidote infusions were needed.

Economic information about uterine cervix cancer disease management serves many potential audiences. We studied the non-prespecified endpoint of triapine cost on treatment compliance in order to inform decisions about health care resource allocation and potential use of an oral triapine formulation. Most cancer drug cost studies employ a two-step procedure—first collecting resource use, and second, collecting unit pricing to arrive at a total estimated cost. We did this here by accounting for intravenous triapine infusion costs, physician visits, monitoring for clinical disease progression, and nursing or regulatory procedure or time expenses. Now that there is an oral triapine formulation in clinical use, future triapine trials might consider comparative analysis of the two formulations in terms of uterine cervix cancer disease effects and cost.

Limitations of this pilot, open-label randomized phase II trial include the small sample size, which limits our assessment of clinical outcomes like overall survival or metabolic complete response; potential investigator bias in assessing the rate of clinical complete response and progression-free survival; and the slight imbalance in disease stage and disease histopathology—favoring the cisplatin-radiotherapy group over the triapine group. Finally, multiple interim analyses were conducted, with an amendment implemented, to assess the need for and design of a phase III trial.

In conclusion, despite its limitations, this randomized phase II study provides proof-of-concept that the combination of triapine-cisplatin-radiotherapy improves metabolic complete responses with a favorable safety profile in women with advanced-stage uterine cervix or vaginal cancers. On the basis of these results, a phase III trial of triapine-cisplatin-radiotherapy in women with advanced-stage uterine cervix or vaginal cancers, adequately powered to study overall survival and progression-free survival, is being conducted (NCT02466971).

## Data Availability Statement

All datasets generated for this study are included in the manuscript/supplementary files.

## Ethics Statement

These trials were carried out in accordance with the recommendations of the National Cancer Institute Cancer Therapy Evaluation Program as well as the coordinating center at Case Western Reserve University and University Hospitals of Cleveland (Cleveland, OH, USA). All patients gave written informed consent in accordance with the Declaration of Helsinki and the guidances of local participating Institutional Review Boards.

## Author Contributions

CK, SA, KM, HC, and SI contributed to the collection and review of trial data, its analysis and authentication, and the writing and approval of this manuscript.

### Conflict of Interest

The authors declare that the research was conducted in the absence of any commercial or financial relationships that could be construed as a potential conflict of interest.
